# Setting targets leads to greater long‐term weight losses and ‘unrealistic’ targets increase the effect in a large community‐based commercial weight management group

**DOI:** 10.1111/jhn.12390

**Published:** 2016-06-14

**Authors:** A. Avery, S. C. Langley‐Evans, M. Harrington, J. A. Swift

**Affiliations:** ^1^ School of BiosciencesUniversity of NottinghamNottinghamUK; ^2^ Slimming WorldDerbysUK

**Keywords:** setting targets/goals, community weight loss

## Abstract

**Background:**

Setting personal targets is an important behavioural component in weight management programmes. Normal practice is to encourage ‘realistic’ weight loss, although the underlying evidence base for this is limited and controversial. The present study investigates the effect of number and size of weight‐loss targets on long‐term weight loss in a large community sample of adults.

**Methods:**

Weight change, attendance and target weight data for all new UK members, joining from January to March 2012, were extracted from a commercial slimming organisation's electronic database.

**Results:**

Of the 35 380 members who had weight data available at 12 months after joining, 69.1% (*n *=* *24 447) had a starting body mass index (BMI) ≥30 kg m^–2^. Their mean (SD) weight loss was 12.9% (7.8%) and, for both sexes, weight loss at 12 months was greater for those who set targets (*P *< 0.001). Those that set ≥4 targets achieved the greatest loss (*P *<* *0.001). The odds ratio for weight loss ≥10% at 12 months was 10.3 (95% confidence interval = 9.7–11.1, *P *<* *0.001) where targets had been set compared to none. At the highest quintile of target size, the size of the first target explained 47.2% (*P *<* *0.001) of the variance in weight loss achieved at 12 months. The mean (SD) BMI reduction in those with a target >25% was 7.6 (4.0) kg m^–2^. A higher percentage of obese members did not set targets (*P *<* *0.001) compared to those with a BMI <30 kg m^–2^.

**Conclusions:**

Much of the variance in weight loss achieved in this population was explained by the number of targets set and the size of the first target. Although obese people were less likely to set targets, doing so increased the likelihood of achieving clinically significant weight loss and, for some ‘unrealistic’ targets, improved the results.

## Introduction

Behaviour change strategies are perceived as important components of the underlying treatment for obesity, namely lifestyle modification through diet and exercise 1. The setting of targets and goals is considered to be an important behavioural change technique 2. Both the Centers for Disease Control and Prevention 3 and the National Institute for Health and Care Excellence 4 recommend that an intervention should encourage individuals to set a weight‐loss target of 5–10% of initial body weight. This has been associated with clinically significant health benefits 5, 6 and is described as ‘realistic’ 4. More recent National Institute for Health and Care Excellence (NICE) guidance suggests that a 3% weight loss is desirable and should be the aim for a 12‐week intervention 1. However, health improvement is only one reason for people wanting to lose weight and maintain weight loss. Physical appearance, social factors such as social pressure and events, improving self‐esteem, energy levels and work performance can also motivate individuals to lose weight and maintain a healthier weight 7, 8.

Weight‐loss targets, goals and expectations are all described as motivators for weight loss 4, although the terms are often confused and used interchangeably, both in practice and in the literature. A weight‐loss target or goal may be defined as the total amount of weight an individual would like to lose 9, whereas expectations are more realistic than targets and should ideally be fluid and change as weight loss occurs 10. Clients will inevitably come into a weight‐loss programme with an idea of the amount of weight that they are aiming to lose. These weight‐loss targets are important because they regulate behaviour by affecting attention, decisions, effort and task persistence 11. They energise and direct behaviour 12, and create the framework through which the behaviour is perceived and evaluated 13.

Evidence suggests that effective diet and exercise modification interventions over a 12–24 week period can result in a weight loss of 5–10% of initial body weight 14 and yet this is unsatisfactory to many obese individuals 15. Weight‐loss targets are often much higher than recommended and influenced by many individual factors, including baseline body mass index (BMI) 16 and sex 17. Weight‐loss targets and expectations are also influenced by the environment, with higher targets in clinical compared to community settings 18.

Targets set by clients are often much higher than what is actually achieved^16^, which has led to high targets being considered ‘unrealistic’ 4 and a cause for concern. The ‘false hope’ syndrome/hypothesis 19 suggests that very ambitious targets relating to weight loss are less likely to be met, and that the subsequent failure will to lead to disappointment, dissatisfaction, decreased effort and relapse. However, evidence demonstrates that non‐attainment of goals does not necessarily stop successful weight losers from maintaining their weight loss 9, 20. With many individuals being more satisfied by smaller weight losses than they expected, it is suggested that weight‐loss goals become less important in the long‐term 14, 21 and maintenance may become easier 22. A recent meta‐analysis concluded that there was no empirical evidence to suggest that setting realistic goals led to greater weight loss, or that unrealistic goals had any negative impact on weight loss 6. Others have gone further and suggested that higher targets may be motivational to some participants who wish to avoid the feeling of disappointment 9. A review looking at the effect of expectations on weight‐loss outcomes concluded that higher targets may lead to higher weight loss at 6–12 months 14.

Indeed, Locke & Latham 11 suggest that goals act in an energising capacity and setting higher targets results in greater effort being made, resulting in a better performance than a lower set target. In addition, goals have an impact on the level of persistence, again with harder targets leading to prolonged effort. Setting realistic targets is claimed to be one of the seven myths about current obesity treatment with insufficient evidence to support the practice 23.

Thus, there is much debate as to the effect that weight‐loss targets have on long‐term weight loss. In the present study, their effect within a large community‐based, commercial weight management group that positively encourages target weight setting was considered to provide further fuel for the debate. The aim was to consider whether self‐imposed target setting predicts weight loss at 12 months in group members with an initial BMI ≥30 kg m^–2^. Three aspects of targets were investigated as predictors: (i) whether target setting was reported by the group member or not; (ii) the number of targets set over the 12‐month data collection period; and (iii) the size of the first target set. It was hypothesised that setting weight‐loss targets leads to a greater amount of weight loss in the long‐term (12‐month period) and significantly more members reaching ≥10% weight loss. For those who do set targets, it was also hypothesised that larger targets and setting a greater number would lead to greater weight loss over 12 months.

## Materials and methods

Slimming World (SW) is a UK‐based commercial weight management programme meeting the NICE (2014) guidance for programme content and constant efforts are made to evaluate and thus improve the support offered to members. SW weekly groups are held throughout the UK and members are further supported by a magazine and website. At SW, weight‐loss targets are referred to as personal achievement targets. Members are strongly encouraged to set their own weight‐loss target, although it is not compulsory and the health benefits of losing 10% initial weight are emphasised. The trained group facilitator may offer advice if requested and will ensure that the target does not lead to a weight below the healthy range. Those who choose to do so have the freedom to set interim and final weight targets, which can be fluid, to suit their own requirements.

Data on new members aged ≥18 years and not pregnant, who joined between January 2012 and March 2012 inclusive, were extracted from the SW electronic database. Data were collected for all members up to either their leaving the group or, for members still attending, up to September 2013. Data available in the SW database were electronically collected from registration forms and group meetings. Members were weighed at the group in light clothing without shoes using calibrated scales (Seca Ltd, Birmingham, UK) to the nearest 200 g. Personal information on each new member included date of recruitment, date of birth, region, sex, self‐reported height, number of attendances, initial weight and weight at 12 months. In addition, any reported interim/final weight‐loss targets and the date of target achievement were also recorded. Names and membership numbers were removed from the database to ensure the anonymity of participants. Ethical approval for the secondary data analysis was approved through the School of Sociology and Social Policy, University of Nottingham.

### Data screening

**Figure 1:**
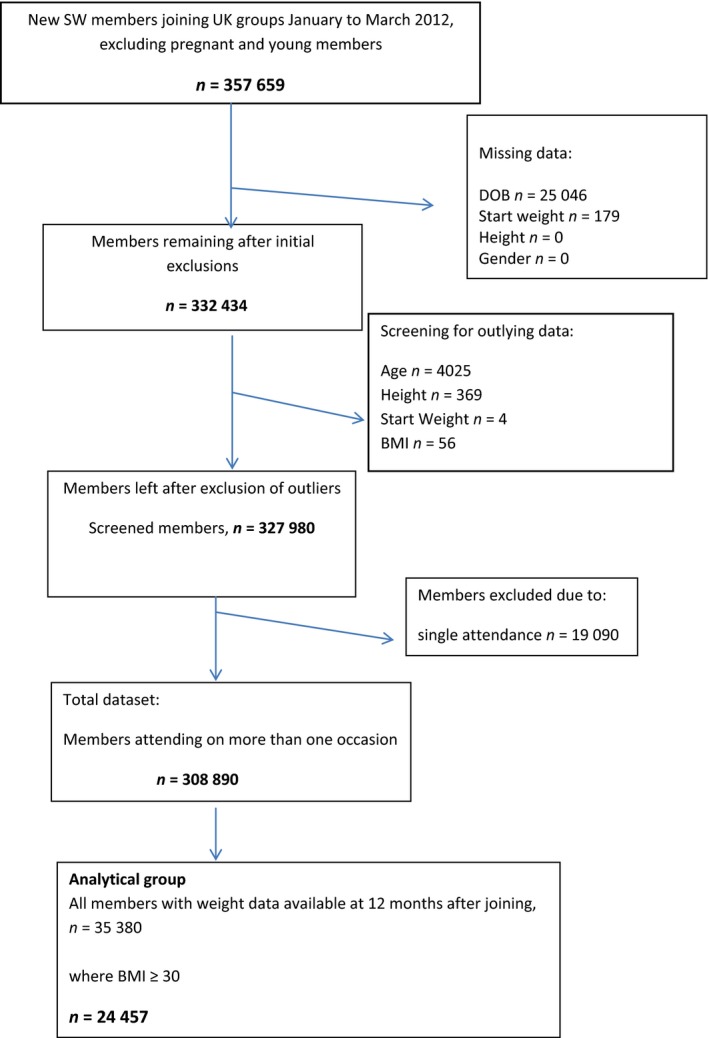
Flowchart of data screening process. SW, Slimming World; DOB, date of birth; BMI, body mass index.

Any non‐UK members within the database were removed, as were any members who only attended the initial group meeting or for whom data on any variable were missing (Fig. 1). Age at baseline was calculated for each member based upon their date of birth and the date of recruitment. Initial BMI and BMI at 12 months, respectively, was calculated by dividing initial weight and weight at 12 months (converted to kg) by the square value of self‐reported height (m). Initial BMI was categorised into normal weight (20–24.9 kg m^–2^), overweight (25–29.9 kg m^–2^), obese (30–39.9 kg m^–2^) and morbidly obese (>40 kg m^–2^). Weight change at 12 months is reported as a percentage of the start weight. The targets achieved are reported as a percentage of the starting weight. Weeks to achieve the first target were calculated from the difference between date of achievement and date of recruitment. Participants were categorised as ‘target set’ and ‘non‐target set’ members based upon whether they had target weights reported. Outliers were screened out using standard SW parameter checks: members were excluded if any of the following applied <18 and >80 years, height <1.35 and >2.1 m, start weight <36 and >273 kg or start BMI <20 and >90 kg m^–2^. Finally, data were screened for any abnormal weight changes >70 kg or > 50% weight loss or >10% weight gain. The original dataset of 376 186 members extracted generated a useable dataset of 308 890 individuals (Fig. 1). An analytical group was then created comprising members from this dataset with 12‐month weight data available and who had an initial BMI ≥30 kg m^–2^. This resulted in an analytical sample of 24 447 individuals (Fig. 1) used to test the study hypotheses.

### Statistical analysis

All statistical analysis was carried out in spss, version 22.0 (IBM Corp., Armonk, NY, USA). All continuous variables were tested for normality as determined by the level of skew and kurtosis. Unless stated otherwise, continuous data are Gaussian and are described using the mean (SD) and bivariate analyses were conducted using independent samples *t*‐tests and Pearson's correlation coefficient. Binominal data were described using frequencies and bivariate analyses were conducted using a chi‐squared test. Data are reported as the mean (SD).

To test the hypothesis that setting weight‐loss targets leads to a greater amount of weight loss in the long‐term (12‐month period), ‘target set’ members and ‘non‐target set’ members in the analytical group were analysed using one‐way analysis of variance (with post‐hoc Bonferroni correction). To test the hypothesis that the size of the first target leads to a greater amount of weight loss in the long‐term (12‐month period), the population was divided into quintiles based upon the size of the first target. To investigate the relative importance of the size of the first target and the number of targets as predictors of percentage weight change at 12 months, stepwise linear regression analysis was performed using adjusted *r* ^2^ values and standardised coefficients (β values) to determine the level of significance. Collinearity statistics were used to determine the level of tolerance. Finally, to determine the predictors of a ≥10% weight loss at 12 months, odds ratios (OR) and corresponding 95% confidence intervals (CI) were determined in the analytical sample as a whole, and within each quintile of the size of the first target.

## Results

### Characteristics of ‘target set’ and ‘non‐target set’ members in the total data set

The mean (SD) initial BMI for the total data set (*n *= 308 890) was 33.1 (6.39) kg m^–2^ and the mean (SD) age at joining was 43.1 (13.6) years. Some 46.6% (*n *= 143 940) were ‘target set’ members and 53.4% (*n *= 164 950) were ‘non‐target set’ members. The majority of members in the total data set were female: 95.2% in the ‘target set’ group and 95.9% in the ‘non‐target set’ group. Members in the ‘target set’ group were significantly older than those in the ‘non‐target set’ [mean (SD) age at joining of 43.4 (13.7) years versus 42.8 (13.5) years, *P* < 0.05]. Members in the ‘target set’ group had a significantly lower initial BMI [mean (SD) 32.1 (6.0) versus 33.8 (6.6) kg m^–2^, *P* < 0.05] compared to members in the ‘non‐target set’ group. A significantly higher percentage of members with normal weight and overweight initial BMI were in the ‘target set’ group (54.9% versus 45.1% in the non‐target set group, *P* < 0.001), whereas a lower percentage of members with an obese or morbidly obese initial BMI (*n *= 197 271) were in the ‘target set’ group (58.1% versus 41.9%, *P* < 0.001). Members in the ‘target set’ group were significantly more likely to attend SW for a longer period [mean (SD) 21.0 (21.4) versus 12.7 (14.4) weeks, *P* < 0.05]. 11.5% (*n *= 35 380) of the total sample had weight data available at 12 months after joining. Significantly more ‘target‐set’ members had a 12‐month weight recorded than ‘non‐target set’ members (18.5% versus 5.4%, *P* < 0.05).

### Analytical group

**Figure 2:**
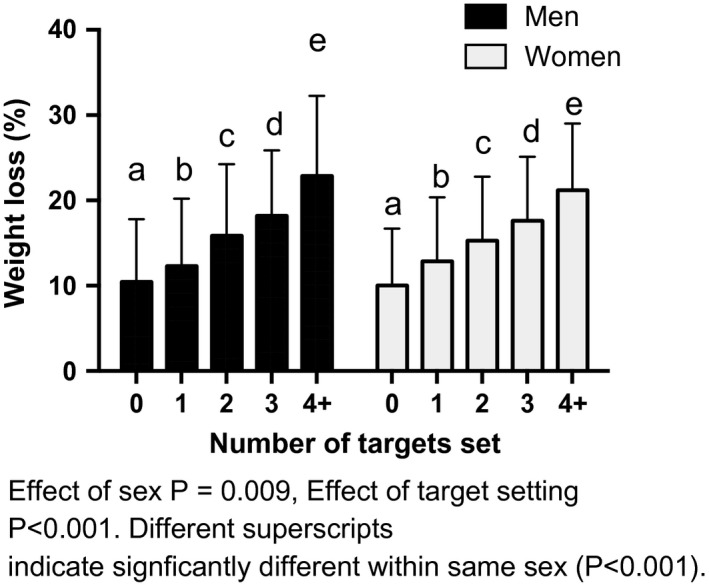
Impact of number of targets set on weight loss at 12 months.

Of the 35 380 members who had weight data available at 12 months after joining, 69.1% (*n *= 24 447) were obese on joining SW. For this analytical group, the mean initial BMI was 37.1 (5.9) kg m^–2^ and age at joining was 47.6 (13.7) years. Mean (SD) weight loss at 12 months was 12.9% (7.8%). 68.2% (*n *= 16 663) were ‘target set’ members and 31.8% (*n *= 7784) were ‘non‐target set’ members (Table 1). Initial BMI, weight loss and BMI at 12 months were influenced by significant interactions of sex and target setting (*P* < 0.001). Both males and females in the ‘target set’ group had lower initial BMI compared to those in the ‘non‐target set’ group, and men had significantly higher initial BMI in both the ‘target set’ and ‘non‐target set’ group (*P* < 0.001). For both sexes, those in the ‘target set’ group were significantly older than those in the ‘non‐target set’ group (*P* < 0.01). There was no significant difference in percentage weight loss at 12 months between men and women in either the ‘target set’ or ‘non‐target set’ group. However, for both sexes, the percentage weight loss at 12 months was significantly greater for members in the ‘target set’ compared to the ‘non‐target set’ group (*P* < 0.001). For both sexes, members in the ‘target set’ group attended more sessions than those who did not (*P* < 0.001) (*n* = 62.8% of ‘target set’ members set 1 target; *n *= 23.7% set 2 targets; *n *= 8.7% set 3 targets; and *n *= 4.8% set 4 or more targets over the 12‐month study period). Those members that set more than four targets over the year achieved significantly greater weight loss (*P* < 0.001) (Fig. 2).

**Table 1:** Characteristics of cohort: comparing target setters and non‐target setters

	No target set		Targets set	
	Men	Women	Men	Women
*n*	474	7310	1383	15 280
Initial weight (kg)	128.3 (25.4)	103.7 (18.7)***	117.9 (20.5)†	96.9 (16.6)***, †
Initial BMI (kg m^–2^)	40.5 (7.1)	38.7 (6.3)***	37.2 (0.7)†	36.3 (5.4)***, †
12 month weight (kg)	114.1 (20.0)	92.9 (16.4)***	100.0 (17.3)†	82.9 (15.3)***, †
12 month BMI	36.0 (5.6)	34.6 (5.6)***	31.5 (5.0)†	31.0 (5.1)***, †
Weight loss (kg)	14.3 (12.2)	10.8 (8.1)***	17.9 (12.5)†	14.0 (8.7)***, †
Weight loss (% initial weight)	10.5 (7.4)	10.0 (6.7)*	14.8 (8.6)†	14.2 (7.8)*, †
Age	48.4 (13.2)	47.5 (13.6)	48.9 (13.3)	47.5 (13.8)**
Number of attendances	60.2 (16.2)	60.6 (15.5)	61.2 (16.0)†	62.5 (15.3)†

Significant effect of sex: *P < 0.05, **P < 0.01, ***P < 0.001.

†Indicates a significant effect of setting target (*P* < 0.001).

All data are shown as the mean (SD). Initial weight, initial body mass index (BMI), weight at 12 months and BMI at 12 months were influenced by significant interactions of sex and target setting (*P* < 0.001).

For the 16 663 ‘target set’ members in the analytical group, the mean (SD) size of the first target was 19.4 (9.0)% weight loss (Table 2). To extend the analysis, the population was divided into quintiles on the basis of the size of first target (Q1: <10.41%; Q2: 10.42–16.35%; Q3: 16.36–20.86%; Q4: 20.87–26.48%; and Q5: >26.48% of weight loss). Members setting the highest first targets were significantly younger than those setting lower first weight‐loss targets. Members setting the highest first targets had significantly higher initial BMI [Q5 mean (SD) 39.4 (5.5) kg m^–2^] than those setting targets in any other quintile [Q1: 36.9 (6.0); Q2: 34.9 (4.9); Q3: 34.8 (4.7); and Q4: 35.7 (4.8) kg m^–2^, respectively, *P* < 0.001]. The mean weight loss at 12 months for all those setting targets was 14.3 (7.9) %. Members setting the highest first target achieved a significantly greater percentage weight loss at 12 months compared to members in other quintiles [13.9 (6.1), 16.1 (7.0) and 19.0% for all of the mean values and sds could we please have mean(sd) and then the unit eg % to be consistent with the other results presented (9.4) for the third, fourth and fifth quintiles, respectively, compared to 11.4 (7.6) and 11.1 (6.0) for the first and second quintiles, *P* < 0.001]. A mean (SD) BMI change of 36.3 (5.5) to 31.0 (5.1) kg m^–2^ was achieved by the group setting targets compared to 38.8 (6.4) to 34.7 (5.6) kg m^–2^ in the group with no targets reported, a difference of 1.1 kg m^–2^ (*P* < 0.001). The higher weight losses achieved at 12 months in those setting higher first weight‐loss targets was reflected in greater BMI changes achieved (Table 2). Members setting higher first targets took significantly longer to achieve their target weight (Table 2). There was no significant difference in terms of the total number of attendances between the quintiles.

**Table 2:** Characteristics of the analytical group including quintiles of initial weight‐loss target

	All subjects	No target set	All with target set	Size of first target 0–10.41% loss (Q1)	Size of first target 10.42–16.35% loss (Q2)	Size of first target 16.36–20.86% loss (Q3)	Size of first target 20.87–26.48% loss (Q4)	Size of first target >26.48% loss (Q5)
*n*	24 447	7784	16 663	3333	3329	3334	3318	3330
% Female	92.4	93.9	91.7	92.9	90.4	91.0	91.7	92.5
Age (years)	47.6 (13.7)	47.6 (13.6)	47.6 (13.8)	48.8 (13.8)	50.8 (13.9)	48.7 (13.7)	46.6 (13.4)	43.0 (12.9)*
Initial BMI (kg m^–2^)	37.1 (5.9)	38.8 (6.4)	36.3 (5.5)†	36.9 (6.0)	34.9 (4.9)**	34.8 (4.7)**	35.7 (4.8)	39.4 (5.5)*
Number of attendances	61.8 (15.5)	60.6 (15.5)	62.4 (15.4)†	61.4 (15.7)	60.5 (15.2)	62.1 (15.1)	63.2 (15.1)	65.0 (15.3)*
Number of targets set			1.6 (1.0)	2.2 (1.2)	1.6 (0.9)	1.5 (0.8)	1.4 (0.8)	1.3 (0.7)*
Weeks to achieve first target	32.9 (20.5)		28.3 (20.5)	18.4 (16.3)	21.5 (17.6)	28.9 (16.9)	34.7 (16.9)	38.2 (16.7)*
Size of first target achieved (% weight loss)	19.4 (9.0)		19.4 (9.0)	8.2 (2.2)	13.8 (1.6)	18.6 (1.3)	23.4 (1.6)	33.1 (5.9)*
Weight loss at 12 months (%)	12.9 (7.8)	10.1 (6.7)	14.3 (7.9)†	11.4 (7.6)**	11.1 (6.0)**	13.9 (6.1)	16.1 (7.0)	19.0 (9.4)*
BMI at 12 months (kg/m^2^)		34.7 (5.6)	31.0 (5.1)†	32.6 (5.5)	30.9 (4.6)	29.9 (4.5)	29.9 (4.8)	31.8 (5.6)*
BMI reduction at 12 months (kg m^–2^)		4.1 (3.0)	5.3 (3.2)†	4.3 (3.2)**	4.0 (2.3)**	4.9 (2.3)	5.8 (2.7)	7.6 (4.0)*

A significant effect across quintiles (*P < 0.001, **P < 0.005) and between quintiles is indicated.

^†^A significant effect of setting target (*P* < 0.001).

Data are shown as the mean (SD). BMI, body mass index.

To identify predictors of percentage weight loss at 12 months, stepwise linear regression was used. Prior to analysis, the data set was screened for missing values and examined for fit between the variables and the assumptions of multivariate analysis. The dependent and independent variables were investigated using bivariate correlation analysis. All independent variables were associated with the dependent variable, although only the size of the first target demonstrated an effect that accounted for more than 30% of the variance (*r* = 0.66). Initial BMI, sex and age accounted for minimal variance (Table S5).

The stepwise linear regression analysis revealed that 65.9% of the variance in weight loss at 12 months was explained by variation in the size of the first weight‐loss target (44.1%), the number of targets set (12.5%), weeks to achieve first target (4.2%) and the total number of attendances (3.3%). Initial BMI, sex and age predicted significant but small percentages of variance (Table 3). Greater weight loss at 12 months was therefore predicted by a greater first target weight loss, more targets being set and greater attendance. Additional stepwise linear regression analyses were conducted on members categorised by the size of the first target, divided into quintiles. Across each of the four lower quintiles, the number of targets set predicted the greatest amount of variance in percenatge weight loss at 12 months (Q1: 15.9%; Q2: 26.4%; Q3: 24.0%; and Q4: 18.8%, respectively, *P* < 0.001). Only at the highest quintile of first target was the size of the first target a significant predictive variable, predicting 47.2% of the variance, with the number of targets set predicting 3.5%. The odds of achieving ≥10% weight loss at 12 months were greater for the ‘target set’ group compared to the ‘non‐target set’ group and were progressively greater with an increasing size of initial weight‐loss target compared to a non‐target set member (Table 4).

**Table 3:** Percentage weight loss at 12 months predictive variables using a stepwise linear regression model

Step	Predictors	Adjusted *r* ^2^	*r* ^2^ change	*F* change	*P*
1	Size of first target	0.441	0.441	7054.296	0.000
2	Size of first target and number of targets set	0.566	0.125	2570.171	0.000
3	Size of first target, number of targets set and weeks to achieve first target	0.608	0.042	960.075	0.000
4	Size of first target, number of targets set, weeks to achieve first target and total attendances	0.641	0.033	825.996	0.000
5	Size of first target, number of targets set, weeks to achieve first target, total attendances and starting BMI	0.657	0.016	413.126	0.000
6	Size of first target, number of targets set, weeks to achieve first target, total attendances, starting BMI and sex	0.659	0.002	40.372	0.000
7	Size of first target, number of targets set, weeks to achieve first target, total attendances, starting BMI, sex and age	0.659	0.000	8.997	0.003

BMI, body mass index.

**Table 4:** Odds ratio (OR) with respecct to achieving ≥10% weight loss at 12 months compared to not setting a target weight

	OR value	95% CI	*P*
All setting a target	10.3	9.7–11.1	<0.001
Q1	1.3	0.9–1.4	<0.001
Q2	1.5	1.3–1.6	<0.001
Q3	3.4	3.1–3.7	<0.001
Q4	4.5	4.1–5.0	<0.001
Q5	4.5	4.1–4.9	<0.001

CI, confidence interval; Q, quintile.

## Discussion

The maintenance of extensive records by a major commercial slimming organisation presents an opportunity to conduct an analysis of a large community sample. Our analysis of a predominantly female population shows that if people with a BMI ≥30 kg m^–2^ maintained attendance of a weight management group, then they were likely to achieve a clinically significant weight loss (≥10% weight loss) at 12 months irrespective of whether they set targets or not. This indicates that maintaining engagement through attendance is very important within this type of weight‐loss setting. The mean weight loss achieved in the present study is very similar to that reported by Lavin *et al*. 24, where 45 395 ‘high‐engagers’ from a separate SW data set achieved a mean 13.2 (7.4)% weight loss at 12 months and thus this should be considered as a normal weight‐loss outcome in slimmers accessing and engaging with community support programmes. In a randomised controlled trial where 377 adults were referred to community Weight Watchers groups, 33% achieved a weight loss greater than 10% at 12 months 25. The reality is that, particularly for the morbidly obese patient, they will need to achieve a weight loss greater than 5–10% to maximise the clinical benefits gained from weight loss.

Although obese and younger people were less likely to set weight‐loss targets, those that did were significantly more likely to achieve a greater weight loss at 12 months than those who did not. Among the obese population in this sample, those who set targets were 10 times more likely to be at least 10% lighter at 12 months. Setting a higher first weight‐loss target, in the range of 20–30% of initial weight, was associated with further improvement in weight‐loss outcomes, although the actual weight loss was nearer 20% at 12 months, equating to a BMI mean reduction of 7.6 kg m^–2^ in those with a target >25%. The combination of the number of targets set and the size of the first target predicted much of the variance seen in weight‐loss outcomes at 12 months irrespective of age, sex, number of attendances and starting BMI, although relationships between all of the variables are seen to some extent. It was observed that, when initial BMI was ≥30 kg m^–2^, those setting high first weight‐loss targets (>26.5%) were younger and heavier at the time of joining the weight management group. This group were 4.5 times more likely to lose ≥10% of their starting weight than those not setting a target. The data suggests that obese people could either set a greater number of smaller weight‐loss targets or choose a higher first weight‐loss target achieved over a longer time, aiming to achieve a clinically beneficial weight loss and healthier BMI at 12 months. Although the findings of this analysis support the benefits of setting targets as part of a behavioural strategy to improve weight‐loss outcomes, the reported data challenge the belief that these targets should be realistic as defined by a 5–10% weight loss. Four‐fifths of those who set a target were aiming for a greater weight loss than this and were still engaged with the weight‐loss programme at 12 months, with clinically beneficial weight losses.

The present study, with a larger study population, supports and builds on the findings of De Vet *et al*. 18, where, in a nonclinical sample, new year's weight‐loss targets of 13.6% were reported, with approximately two‐thirds of the 447 participants setting targets that exceeded 10%. It was suggested that not providing any moderating guidelines on setting weight‐loss targets may yield positive attainment outcomes and that the amount of weight loss individuals strive for may lead to more effort. Casazza *et al*. 23 cited setting realistic targets to be one of the seven myths about current obesity treatment with insufficient evidence to support the practice. The present study further fuels this claim and suggests that national guidance on weight management needs to be reviewed with the emphasis on setting realistic targets being removed. For a number of people, setting ambitious weight‐loss targets will be motivating and meaningful. Houser‐Marko & Sheldon^26^cite higher‐level targets as being more self‐relevant and holistic, providing a sense of direction and purpose.

The present study adds to current knowledge by examining the association between the number of targets set and the size of the first weight‐loss target and weight loss‐outcomes at 12 months for both men and women. Undeniably, the group of participants setting the highest weight‐loss targets (Q5) achieved the greatest weight loss at 12 months, although the data also suggest that, for both sexes, there will be some individuals who may benefit from setting a greater number of smaller weight‐loss targets and the balance between the two approaches may need to be fluid according to personal circumstances 27. Crawford & Glover^14^, in their review, highlighted the lack of published evidence examining the fluidity of target setting in relation to weight‐loss outcomes, with data particularly lacking for men.

One of the definitions for weight‐loss maintenance described by Elfhag & Rossner^28^ is ‘achieving an intentional weight loss of at least 10% and maintaining this body weight for at least one year’. In their review, they identified successful weight maintenance being associated with, along with other factors, more initial weight loss, reaching a self‐determined target weight and social support. One of the benefits of enrolling with a commercial slimming organisation is the social support offered to all members and this may facilitate the higher individual target weights to be set, leading to improved weight loss at 12 months 29.

The major strength of the present study is that it presents data from a large community sample of both men and women, albeit with a small percentage of men that is representative of enrolment and attendance at commercial slimming organisations 24, 25. However, the study size was sufficiently large to detect any outcome differences between the sexes. A limitation is that the data set was unfortunately incomplete, with 12‐month weight being not recorded for a significant number of people in the original sample. This most likely arose because members may have had successful or unsuccessful weight‐loss journeys and were longer attending the slimming group at 12 months. It is also possible that some people may have left and re‐joined after a more than 4‐week break and this is not captured in the data analysis.

With the finding that 12‐month weight change is influenced by target setting, it would be of future interest to perform analyses that consider weight change over longer periods (24 or 36 months) aiming to determine whether the successful weight losses are maintained. The time‐frame for data collection precludes such analysis at present. Other, smaller hospital‐based studies suggest that achieving weight‐loss targets is associated with the maintenance of weight loss over a 24‐month period 29. The present study did not consider the relationship between weight‐loss expectations and target setting and no data were collected to establish whether the people in the sample were satisified with the weight losses that they achieved, or whether they hoped to achieve their targets in a shorter time period. It would be of benefit to know why the study population did or did not set targets, as well as what influenced this decision, because such information could be used to refine current guidance or programmes that assist in weight loss. In particular, it would be beneficial to know more about what influenced some people to set very high targets (>20% weight loss), which appeared to be a successful strategy for achieving greater weight loss over 12 months. Further studies are required to answer some of these unknowns.

To date, no study based in a community setting has investigated weight‐loss targets on this scale and detail, with both the size of the target and the number of targets being reported. Obese people are less likely to self‐impose targets but, if they do, the more targets they set, they more likely they are to achieve a greater weight loss at 12 months. Setting an ambitious first weight‐loss target is going to further improve weight‐loss outcomes. This contradicts current national guidance and it is proposed that the current approach to set ‘realistic’ weight‐loss targets is indeed a myth and should be questioned and reviewed. The combination of the size of the first target and the number of targets set predicts much of the variance seen in weight‐loss outcomes at 12 months, irrespective of baseline BMI, sex and age.

**Conflicts of interest, source of funding and authorship**

AA, alongside her academic position at the University of Nottingham also holds a consultancy position at Slimming World.

This research received no specific grant from any funding agency in the public, commercial, or not‐for‐profit sectors.

AA, MH and JAS designed the study. AA, SLE and JAS completed the data analysis. AA, SLE and JAS prepared and critically reviewed the manuscript.

## Supporting information


**Table S1.** Means, SDs and bivariate (Pearson) correlations between the main variables of the study. Note all correlations are significant at the *P* < 0.01, except where indicated by an asterisk (*) where *P* < 0.05, and no correlation was observed between age and the total number of contacts^$^.

**Table S2.** The predictors of weight loss at 12 months for each size of first target quintile group using stepwise linear regression.
