# Supplementary material for: Setting targets leads to greater long‐term weight losses and ‘unrealistic’ targets increase the effect in a large community‐based commercial weight management group
Source: J Hum Nutr Diet. 2016 Jun 14;29(6):687–96. doi: 10.1111/jhn.12390 (PMC5111772; doi:10.1111/jhn.12390)
Supplement: Supplementary file 1 — Table S1. Means, SDs and bivariate (Pearson) correlations between the main variables of the study. Note all correlations are significant at the P < 0.01, except where indicated by an asterisk (*) where P < 0.05, and no correlation was observed between age and the total number of contacts$. [file JHN-29-687-s001.docx]

|  | *1* | *2* | *3* | *4* | *5* | *6* | *7* | *8* |
| --- | --- | --- | --- | --- | --- | --- | --- | --- |
| % weight loss at 12 months (1) | - | 0.66 | 0.20 | 0.26 | 0.14 | -0.16 | 0.21 | 0.02* |
| Size of first target (2) | - | - | -0.23 | 0.14 | 0.58 | -0.11 | 0.14* | 0.03 |
| No of targets set (3) | - | - | - | 0.11 | -0.38 | -0.08 | 0.11 | -0.08 |
| Total number of contacts (4) | - | - | - | - | 0.24 | 0.05^$^ | 0.12 | 0.013* |
| No of weeks to achieve first target (5) | - | - | - | - | - | 0.04 | -0.02* | -0.07* |
| Age (6) | - | - | - | - | - | - | -0.10 | 0.02 |
| Starting BMI kg/m^2^ (7) | - | - | - | - | - | - | - | 0.05 |
| Sex (8) |  |  |  |  |  |  |  |  |
| Mean | 12.9 | 19.4 | 1.1 | 61.8 | 32.9 | 47.6 | 37.1 |  |
| Standard Deviation | 7.79 | 9.03 | 1.08 | 15.45 | 20.50 | 13.74 | 5.88 |  |

**Supplementary Table 1**; Means, standard deviations and bivariate (Pearson) correlations between study’s main variables

Note all correlations significant at the 0.01 level apart from * where significance is at 0.05 level and no correlation observed between age and total number of contacts^$^.
