# Supplementary material for: Setting targets leads to greater long‐term weight losses and ‘unrealistic’ targets increase the effect in a large community‐based commercial weight management group
Source: J Hum Nutr Diet. 2016 Jun 14;29(6):687–96. doi: 10.1111/jhn.12390 (PMC5111772; doi:10.1111/jhn.12390)
Supplement: Supplementary file 2 — Table S2. The predictors of weight loss at 12 months for each size of first target quintile group using stepwise linear regression. [file JHN-29-687-s002.docx]

Supplementary Table 2; the predictors of weight loss at 12 months for each size of first target quintile group using step-wise linear regression.

|  | All subjects | No target set | All with target set | Size of first target, (Q1) | Size of first target, (Q2) | Size of first target, (Q3) | Size of first target, (Q4) | Size of first target, (Q5) |
| --- | --- | --- | --- | --- | --- | --- | --- | --- |
| Size of first target weight | 44.1 |  | 44.1 | 7.2 | 4.6 | 7.2 | 10.0 | 47.2 |
| No of targets set | 12.5 |  | 12.5 | 15.9 | 26.4 | 24.0 | 18.8 | 3.5 |
| Weeks to achieve first target | 4.2 |  | 4.2 | 5.9 | 4.7 | 6.2 | 7.2 | 16.9 |
| No of attendances | 3.3 | 3.2 | 3.3 | 5.2 | 5.4 | 4.5 | 4.0 | 0.9 |
| Starting BMI kg/m^2^ | 1.6 | 7.6 | 1.6 | 3.1 | 1.7 | 2.3 | 1.9 | 0.3 |
| sex | 0.2 | 0 | 0.2 | 0 | 0 | 0.3 | 0 | 0 |
| age | 0 | 1.2 | 0 | 0.3 | 0 | 0 | 0 | 0 |
| **Total %variance predicted** | **65.9** | **12.0** | **65.9** | **37.6** | **42.8** | **44.5** | **41.9** | **68.8** |
